# Prediction of Quantum Anomalous Hall Insulator in half-fluorinated GaBi Honeycomb

**DOI:** 10.1038/srep31317

**Published:** 2016-08-10

**Authors:** Sung-Ping Chen, Zhi-Quan Huang, Christian P. Crisostomo, Chia-Hsiu Hsu, Feng-Chuan Chuang, Hsin Lin, Arun Bansil

**Affiliations:** 1 Department of Physics, National Sun Yat-Sen University, Kaohsiung 804, Taiwan; 2 Centre for Advanced 2D Materials and Graphene Research Centre, National University of Singapore, Singapore 117546; 3 Department of Physics, National University of Singapore, Singapore 117542; 4 Department of Physics, Northeastern University, Boston, Massachusetts 02115, USA

## Abstract

Using first-principles electronic structure calculations, we predict half-fluorinated GaBi honeycomb under tensile strain to harbor a quantum anomalous Hall (QAH) insulator phase. We show that this QAH phase is driven by a single inversion in the band structure at the Γ point. Moreover, we have computed the electronic spectrum of a half-fluorinated GaBi nanoribbon with zigzag edges, which shows that only one edge band crosses the Fermi level within the band gap. Our results suggest that half-fluorination of the GaBi honeycomb under tensile strain could provide a new platform for developing novel spintronics devices based on the QAH effect.

Topological materials are attracting intense current interest in search of new materials platforms for energy-efficient device applications^1^,^2^,^3^,^4^,^5^. These include two-dimensional (2D) topological insulators (TIs), also called quantum spin Hall (QSH) insulators^3^,^6^,^7^, topological superconductors^2^, topological crystalline insulators (TCIs)^8^,^9^, and quantum anomalous Hall (QAH)^5^,^10^ insulators, among others. At the heart of these developments is the discovery of the quantum Hall effect, which led to the realization of the topological nature of the underlying electronic structures and the associated dissipationless spin-transport around the edges of a 2D insulator under a strong magnetic field^11^,^12^.

QAH effect is similar to the quantum Hall effect except that in the case of the QAH the driving magnetic field is generated internally in the material through spin-orbit coupling (SOC), while in the more conventional quantum Hall case the magnetic field is applied externally^13^. The existence of a QAH insulator phase was first inferred via an analysis of a tight-binding model on a honeycomb lattice^14^,^15^, where the Hall conductance was found to be quantized even in the absence of an external magnetic field^16^. The natural place to search for materials realization of the QAH phase is a ferromagnetic insulator with a topologically non-trivial band structure^17^,^18^,^19^. A strong enough magnetization in the QSH phase could then perhaps drive the material into the QAH phase^17^.

QAH effect was first realized in magnetically doped TI thin films^13^,^18^,^20^,^21^,^22^,^23^,^24^, and has been the subject of extensive theoretical studies^19^,^25^,^26^,^27^. In principle, we might expect the QAH effect to be more robust compared to the QSH effect where one needs to be in the submicron regime in order to prevent spin-flip scattering^17^. Notably, quantization can be achieved even at low mobility^17^,^21^. In any event, the fact remains that the presently available QAH materials are constrained to very low temperatures and are based on magnetic doping of thin TI films. There is great need thus to find new QAH materials based on non-magnetic thin films, which would be viable for room temperature applications.

Several thin films of elements of groups IV^28^,^29^ and V^30^,^31^,^32^, which have been predicted to be QSH insulators, have also been predicted to harbor the QAH phase^33^,^34^,^35^,^36^. In contrast, even though a number of alloys of elements of groups III and V have been predicted to support QSH phases^37^,^38^,^39^,^40^,^41^ in freestanding^37^ and functionalized forms^38^,^40^,^41^, the possible existence of QAH phases in these alloys has, to our knowledge, not been explored.

With this motivation, here we examine the possible presence of QAH phases in thin films of GaBi III-V honeycombs using first-principles calculations, including effects of half-fluorination of the films. The Chern numbers in a number of cases are computed to show that the films support the QAH phase over a reasonable range of lattice constant values. We further confirm our results by computing edge states for fluorinated GaBi nanoribbons. Our results imply that on a suitable substrate, which will induce the appropriate tensile strain, the GaBi III-V films could provide a new materials platform for applications based on the QAH effect.

## Results

**Figure 1:**
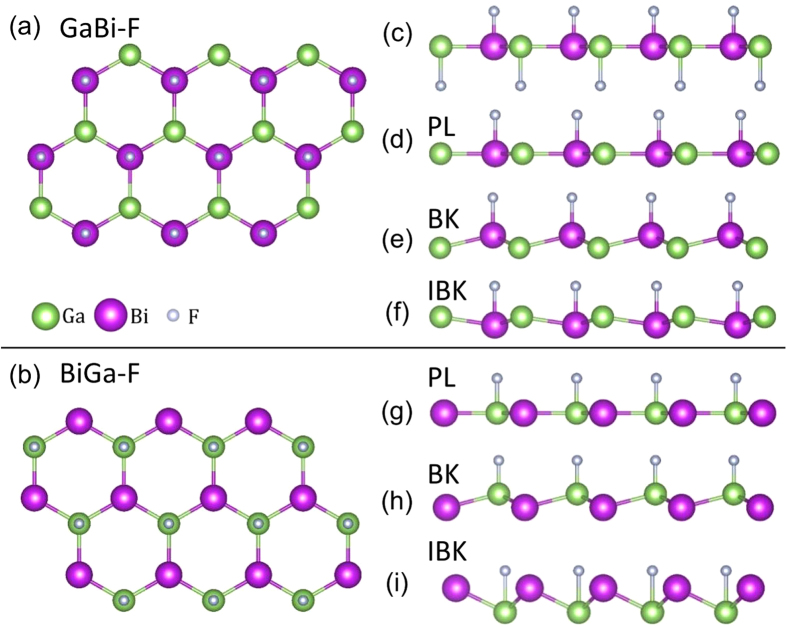
Crystal structure of the GaBi honeycomb with fluorine atoms adsorbed on (**a**) Bi and (**b**) Ga atoms. Side views for different fluorinations of GaBi: (**c**) fully-fluorinated GaBi; (**d**–**f**) Half-fluorination on Bi atoms for a planar (**d**) buckled (**e**), and inversely buckled (**f**) honeycomb; (**g**–**i**) Half-fluorination on Ga atoms for a planar (**g**), buckled (**h**), and inversely buckled (**i**) honeycomb.

Figure 1(a,b) show the top views of the 2D crystal structure of our GaBi honeycomb with fluorine atoms adsorbed on Bi and Ga layer, respectively. Figure 1(c) shows the side view of the fully-fluorinated GaBi. For half-fluorinations, F atoms can be adsorbed either on Bi (denoted by GaBi-F) or on Ga (denoted by BiGa-F). The side-views of different fluorinations of GaBi are shown in Fig. 1(d–i). There are three types of possible configurations: planar (PL), buckled (BK), and inversely buckled (IBK) honeycomb. Here, BK [(e) & (h)] refers to cases where the adsorbed F atom retains the buckling of the original un-passivated GaBi structure^37^,^38^, while IBK [(f) & (i)] refers to the case in which the buckled honeycomb is inverted after half-passivation.

**Figure 2:**
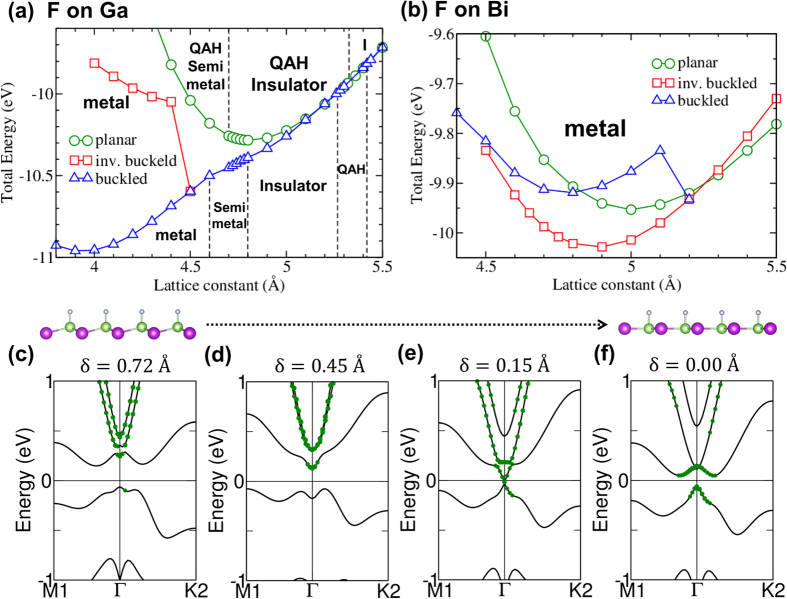
Total energy per unit cell as a function of lattice constant and the associated phases for (**a**) F on Ga [same as BiGa-F in Fig. 1(b)] and (**b**) F on Bi [same as GaBi-F in Fig. 1(a)]. Planar, inversely buckled, and buckled structures are labeled as hollow green circles, red squares, and blue triangles, respectively. (**c**–**f**) Band structures for various values of the buckling distance *δ*, showing the transition from the buckled to the planar honeycomb. *p*_*x*_-orbital contribution is shown (solid green circles).

The computed total energies in Fig. 2 show that half-fluorination of Ga atoms is energetically more favorable than that of Bi atoms. For half-fluorination of Ga, the inversely buckled honeycomb is seen in Fig. 2 to transform into the buckled honeycomb as the lattice constant is increased. In sharp contrast, half-fluorination of Bi, transforms the buckled honeycomb into the inversely buckled honeycomb. By tracking evolution of the band structure and computing the corresponding Chern numbers as a function of strain, we can identify the emergence of topological phases in these films. The resulting phase diagram for half-fluorination on Ga (labeled as BiGa-F) is shown in Fig. 2(a). For the planar case, we obtain a QAH semi-metal for lattice constant (*a*) values below 4.70 Å. PL then yields a QAH insulator between *a* = 4.70 to 5.325 Å and, eventually turns into a trivial insulator above *a* = 5.325 Å. As for the buckled structure, it is a trivial insulator over the range *a* = 4.80–5.26 Å, but it becomes a QAH insulator for *a* = 5.26 to 5.50 Å. The inversely buckled honeycomb (red squares in Fig. 2(a)) is very unstable and transforms into the buckled honeycomb beyond *a* = 4.5 Å. On the other hand, for F adsorbed on Bi atoms, shown in Fig. 2(b), (labeled as GaBi-F), we obtain a metallic state for all configurations. For this reason, for the remainder of this study, we will focus on films with half-fluorination of Ga. Notably, we also find QAH phases for half-hydrogenation and half-halogenation with Cl or Br on Ga (see Figs S1 and S6 in the Supplementary Information).

In order to understand the role of buckling in driving the topological phase transition, we consider in Fig. 2 how the band structure of the film with fixed *a* = 4.80 Å evolves as the buckling distance, *δ*, is decreased from 0.72 Å until the structure becomes planar. The band gap is seen to close at *δ* = 0.15 Å as, see Fig. 2(e). By plotting the *p*_*x*_-orbital contributions (green circles), we can differentiate between the trivial band gap in Fig. 2(c,d) and the inverted band gap in Fig. 2(f). We will return below to show that the Berry curvature near the inverted band gap region assumes large values, yielding a Chern number of −1. For the three aforementioned crystal structures, the topological phase transitions due to strains in the PL, BK and IBK structures are further discussed in Figs S2–S4 in Supplementary Information. In particular, Fig. S3 demonstrates the transition from an insulator to a QAH insulator in the buckled BiGa-F with a critical point at *a* = 5.26 Å. In Fig. S4, the planar honeycomb is seen to go from a QAH semi-metal to a QAH insulator, and to then transition from a QAH insulator to a trivial insulator with increasing lattice constant. In short, for the half-fluorinated and planar GaBi films, we obtain a stable QAH phase over a reasonable range of lattice constants from 4.7 Å to 5.325 Å. Notably, the BiGa-F film at *a* = 4.80 Å exhibits a band gap of 56 meV, but this gap value increases to 105 meV at *a* = 5.50 Å, which is large enough for realizing the QAH phase above room-temperature.

**Figure 3:**
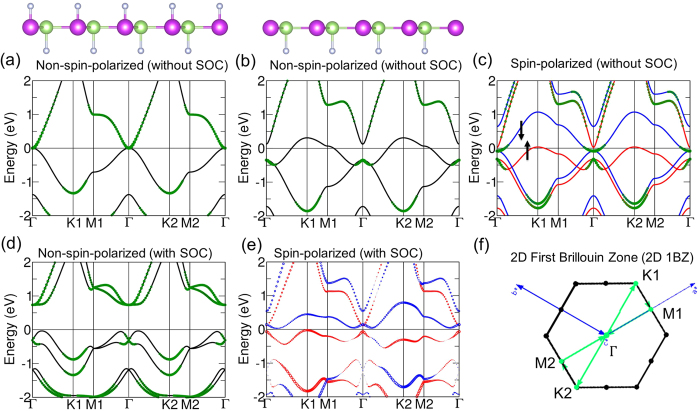
Band structures of planar GaBi films without (top row) and with (bottom row) SOC. (**a**,**d**) Non-spin-polarized bands for a fully-fluorinated film. (**b**,**c**,**e**) Give bands for a half-fluorinated film with F atoms on Ga (BiGa-F) for various cases as indicated at the top of these figures. (**f**) First Brillouin zone with specific high symmetry points labeled. *p*_*x*_-orbital contributions are shown, and are proportional to the sizes of the green circles. Spin-up and spin-down states are represented by red and blue lines, respectively.

Recent first-principles studies have shown that freestanding^37^ and functionalized GaBi^38^,^40^ non-magnetic films can support a robust QSH insulator phase. Half-fluorination or half-hydrogenation of GaBi on Ga (BiGa-F) leads to an intrinsic magnetization strong enough to drive the film into the QAH phase. In this connection, band structures of fluorinated GaBi films in a planar honeycomb are presented in Fig. 3. Fully-fluorinated GaBi (*a* = 4.80 Å) without and with SOC are considered in Fig. 3(a,d), respectively. Fully-fluorinated GaBi is gapless without SOC, but adding SOC in the calculations is seen to open a bulk band gap of ~0.77 eV; we have verified that the film exhibits a QSH phase with Z_2_ = 1. At the equilibrium lattice constant of half-fluorinated planar GaBi (4.80 Å), without SOC, we found the magnetic ground state in the spin-polarized calculations [Fig. 3(c)] to have a lower energy than the nonmagnetic state [Fig. 3(b)]. However, when the SOC was turned on in spin-polarized calculations [Fig. 3(e)], we obtained the QAH phase. Note that in Fig. 3(c,e), the spin-up and spin-down polarized states are marked with red and blue lines/circles, respectively.

Our non-spin-polarized band calculations without the SOC [Fig. 3(b)] show that the material is a zero-gap metal with degenerate states at the Fermi energy at Γ. When spin-polarization is included in the computations [Fig. 3(c)], the exchange field causes the electronic spectrum to split into two sets of bands with different spin polarizations with an intrinsic magnetization of approximately 0.7 *μ*B per unit cell with co-existing gapped spin-up and gapless spin-down dispersions; the spin-down bands continue to be degenerate at Γ. Note that the *p*_*x*_-orbitals (green circles) around Γ of the spin-down bands have become inverted compared to the corresponding bands in Fig. 3(a). Furthermore, the inclusion of SOC leads to an insulating state with a gap (~56 meV), see Fig. 3(e). We thus realize the QAH phase via a *p*_*x*_-orbital inversion in the spin-up band in the presence of an SOC induced band gap. Further plots of *p*_*x*,*y*_-orbital contributions are provided in Fig. S4 of the Supplementary Information.

We have considered effects of half-halogenations of the planar GaBi honeycomb using other elements of the halogen group (Cl, Br, and I). Like F and H atoms, we found more generally that half-halogenations all prefer to adsorb on Ga atoms. At their equilibrium lattice constants both half-chlorinated and half-brominated films are in the QAH phase, whereas half-iodinated films are trivial, although the QAH phase can be induced in half-iodinated films under a compressive strain. Detailed band structures of the halogenated films are given in Fig. S6 of the Supplementary Information.

**Figure 4:**
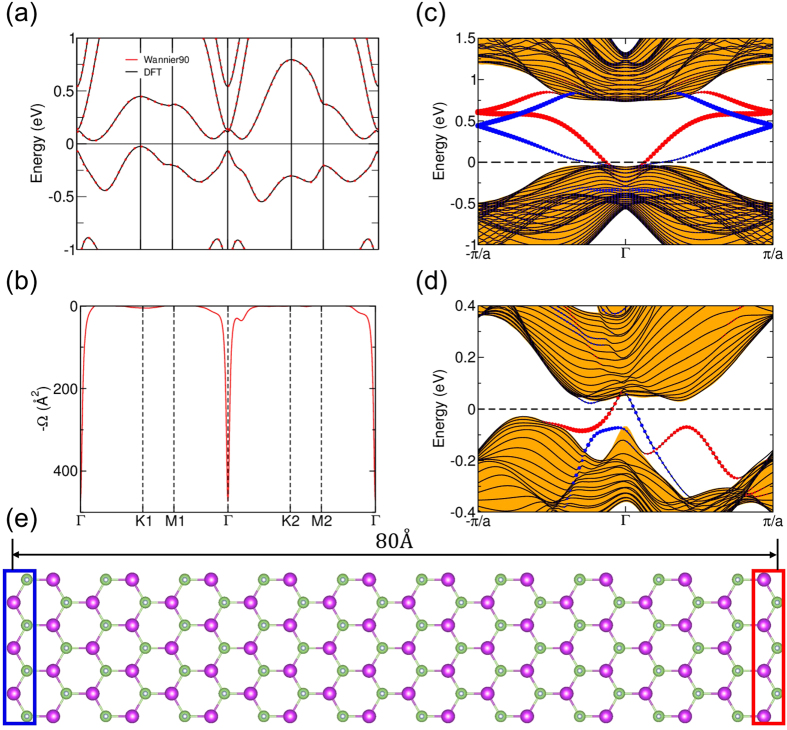
(**a**) Comparison of first-principles (black lines) and tight-binding band structures (red lines) for a GaBi film. (**b**) Computed Berry curvature corresponding to the band structure of panel (**a**). (**c**,**d**) Band structures of a GaBi nanoribbon with zigzag edges of a fully-fluoridated (**c**) and a half-fluoridated (**d**) ribbon. The sizes of blue and red circles are proportional to the contributions of left and right zigzag edges, respectively. Regions with orange filling denote bulk bands. (**e**) Nanoribbon with zigzag edges. *a* = 4.80 Å and width is about 80 Å.

We turn next to discuss the nature of edge states of GaBi nanoribbons. For this purpose, we use a tight-binding Hamiltonian, which is parameterized via Wannier functions, and as Fig. 4(a) shows, our tight-binding model reproduces the first-principles band structure quite well. The Berry curvature of the half-fluorinated GaBi film at a lattice constant of 4.80 Å along a few symmetry directions in the Brillouin zone (BZ) is shown in Fig. 4(b). The value of the Berry curvature is seen to be large around the band inversion region near the Γ point, and when this curvature is integrated over the first BZ, it yields a Chern number of −1 as expected. Chern number is an important quantity for monitoring the QAH effect because the Hall conductivity is proportional to the Chern number. In order to further insight into the nature of the QAH edge state in the half-fluorinated GaBi film as compared to the QSH edge states in a fully-fluorinated GaBi film, we constructed a nanoribbon with zigzag edge as shown in Fig. 4(e). Using our tight-binding model Hamiltonian to calculate the edge states, results for the fully-fluorinated and half-fluorinated nanoribbons are given in Fig. 4. The sizes of blue and red circles are proportional to the contributions of left and right hand side zigzag edges, respectively. Figure 4(c) shows the electronic structure of the fully-fluorinated QSH nanoribbon. The plot exhibits an odd number of band crossings with the Fermi level between *π*/*a* and Γ^38^ and the related time-reversed partners −*π*/*a* and Γ, and thus establishes clearly the existence of helical edge states. In Fig. 4(d), on the other hand, for each side of the half-fluorinated nanoribbon edge, we can see that there is only one chiral edge state connecting the conduction and valence bands, which lies in the middle of the band gap. Note that the number of chiral edge state gives the the absolute value of the Chern number.

In order to assess the robustness of our calculations to the underestimation of the band gap within the GGA, we have computed electronic structures and Berry curvatures of our fully-fluorinated as well as half-fluorinated GaBi films using the hybrid functional HSE06^42^. We find that our GGA-based results in Fig. 2 correctly capture the evolution of the topological phases with strain, some differences in the exact values of the strain at which various phase transitions occur for different exchange-correlation functionals notwithstanding.

Regarding experimental realization, our predicted half-passivated III-V honeycombs could be realized by growing these honeycombs on a suitable substrate. A recent study^33^ has shown that half-iodinated stanene could be realized in stanene grown on CdTe. In addition, one study^43^ SiC-H utilized ultra-high purity hydrogen etching to modify SiC(0001) into H-passivated SiC which is equivalent to half-hydrogenation, and might be appropriate for half-halogenations more generally. Studies toward realizing GaBi films via Bi-doped GaAs^44^, and related works attempting honeycomb-like InBi on Si(111)^45^, has been also reported. Most recently, another study^46^ explored the growth of TlBi film by depositing Bi on Tl-covered Si(111) surface for which a variety of different surface reconstructions such as, 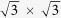 and 4 × 4 for Tl_0.75_Bi_0.25_ and Tl_0.632_Bi_0.368_, respectively, were observed. Our study demonstrates the tunability of the half-halogenated/half-hydrogenated III-V films, which implies a suitable substrate could induce the QAH phase in these III-V films.

## Summary and Conclusions

We have presented an *ab initio* study of the electronic and topological properties of GaBi films functionalized through hydrogenation/halogenation. The presence of a QAH insulator phase is predicted in half-fluorinated GaBi honeycomb under tensile strain. This QAH phase is driven by a single inversion in the band structure at the Γ point. Our computations on a half-fluorinated GaBi nanoribbon with zigzag edges reveal that the edge state spectrum consists of a single chiral band crossing the Fermi level within the band gap. Our study suggests that half-fluorinated GaBi honeycombs could provide a new materials platform for exploiting the QAH effect in spintronics applications.

## Methods

Our first-principles calculations were performed within the framework of the density functional theory (DFT) utilizing the generalized gradient approximation (GGA)^47^,^48^,^49^,^50^,^51^. Projector-augmented-wave (PAW)^52^ wave functions with energy cut-offs of 300 and 400 eV for hydrogenation and fluorination, respectively, were used in the Vienna Ab-Initio Simulation Package (VASP)^53^,^54^. Atomic positions were optimized for each lattice constant value considered until the residual forces were no greater than 10^−3^ eV/Å. Convergence criterion for self-consistency in electronic structure computations was set at 10^−6^ eV. A vacuum layer of at least 30 Å along the *z* direction was used to simulate thin films. A Γ-centered Monkhorst-Pack^55^ grid of 12 × 12 × 1 was used for 2D integrations in the Brillouin zone. Berry curvatures and edge states were calculated based on an effective tight-binding Hamiltonian obtained by using maximally-localized Wannier functions via the WANNIER90 package^56^. In connection with topological properties, Chern number *C* were computed for various cases using^15^,^25^,^57^:


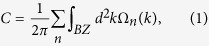


where Ω_*n*_(*k*) is the Berry curvature for the *n*th band^15^,^58^,^59^.

## Additional Information

**How to cite this article**: Chen, S.-P. *et al*. Prediction of Quantum Anomalous Hall Insulator in half-fluorinated GaBi Honeycomb. *Sci. Rep*. **6**, 31317; doi: 10.1038/srep31317 (2016).

## Supplementary Material

Supplementary Information
