# Supplementary material for: Prediction of Quantum Anomalous Hall Insulator in half-fluorinated GaBi Honeycomb
Source: Sci Rep. 2016 Aug 10;6:31317. doi: 10.1038/srep31317 (PMC4979090; doi:10.1038/srep31317)
Supplement: Supplementary Information [file srep31317-s1.pdf]

Supplementary Information for Prediction of Quantum Anomalous Hall Insulator in half-fluorinated GaBi Honeycomb

Sung-Ping Chen,[1,*] Zhi-Quan Huang,[1,*] Christian P. Crisostomo,[1]
Chia-Hsiu Hsu,[1] Feng-Chuan Chuang,[1,†] Hsin Lin,[2,3,‡] and Arun Bansil[4]

[1] *Department of Physics, National Sun Yat-Sen University, Kaohsiung 804, Taiwan*

[2] *Centre for Advanced 2D Materials and Graphene Research Centre, National University of Singapore, Singapore 117546*

[3] *Department of Physics, National University of Singapore, Singapore 117542*

[4] *Department of Physics, Northeastern University, Boston, Massachusetts 02115, USA*

[*] These two authors contributed equally to this work.

[†] fchuang@mail.nsysu.edu.tw

[‡] nilnish@gmail.com

I. Half-hydrogenated GaBi films

Results for GaBi films with half-hydrogenation on (a) Ga (BiGa-H) and (b) Bi (GaBi-H) are presented in Fig. S1. For BiGa-H in Fig. S1(a), the QAH phase appears for planar structure (PL) at lattice constants of 5.08 to 5.34 Å , while for the buckled (BK) honeycomb it appears from 5.30 to 5.50 Å . For half-hydrogenation on Bi in Fig. S1(b), IBK (inversely buckled honeycomb) is energetically favorable and there is no QAH phase. Only metallic states for IBK ($a > 5.00$ Å) and semi-metal states for BK ($a < 5.00$ Å) are found.

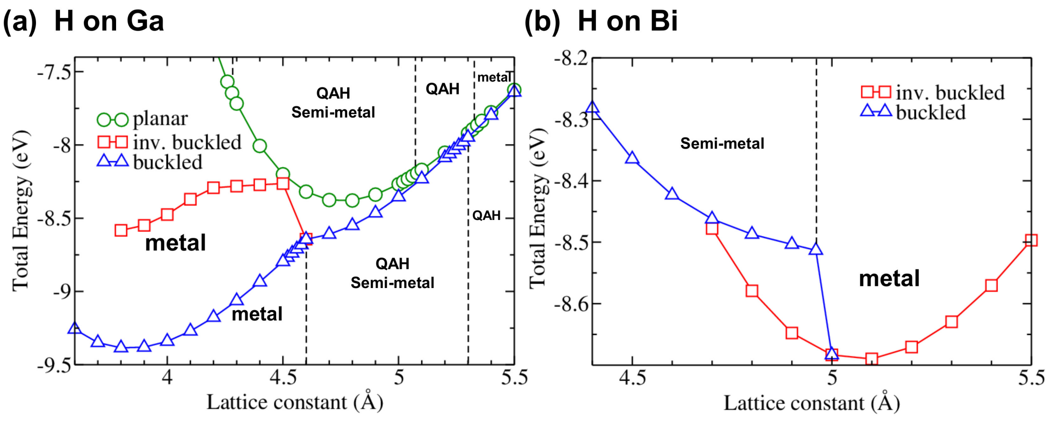

Figure S1. Total energy per unit cell as a function of lattice constant and the associated phases for (a) H on Ga and (b) H on Bi. Planar, inversely buckled, and buckled structures are labeled as hollow green circles, red squares, and blue triangles, respectively.

II. Topological phase transition of buckled to planar BiGa film with half-fluorination on Ga

In order to demonstrate the evolution of the topological phase with buckling, we present in Fig. S2 band structures of BiGa-F films (a =4.80 Å) along two high symmetry lines in the Brillouin zone for various values of the buckling height δ. The corresponding Berry curvatures are also shown. The band structure is seen to transition to that of a QAH insulator with a critical point at δ=0.15 Å. The sharply peaked Berry curvature distribution is due to the band inversion at the zone center, which drives the large associated change in

the Berry curvature.

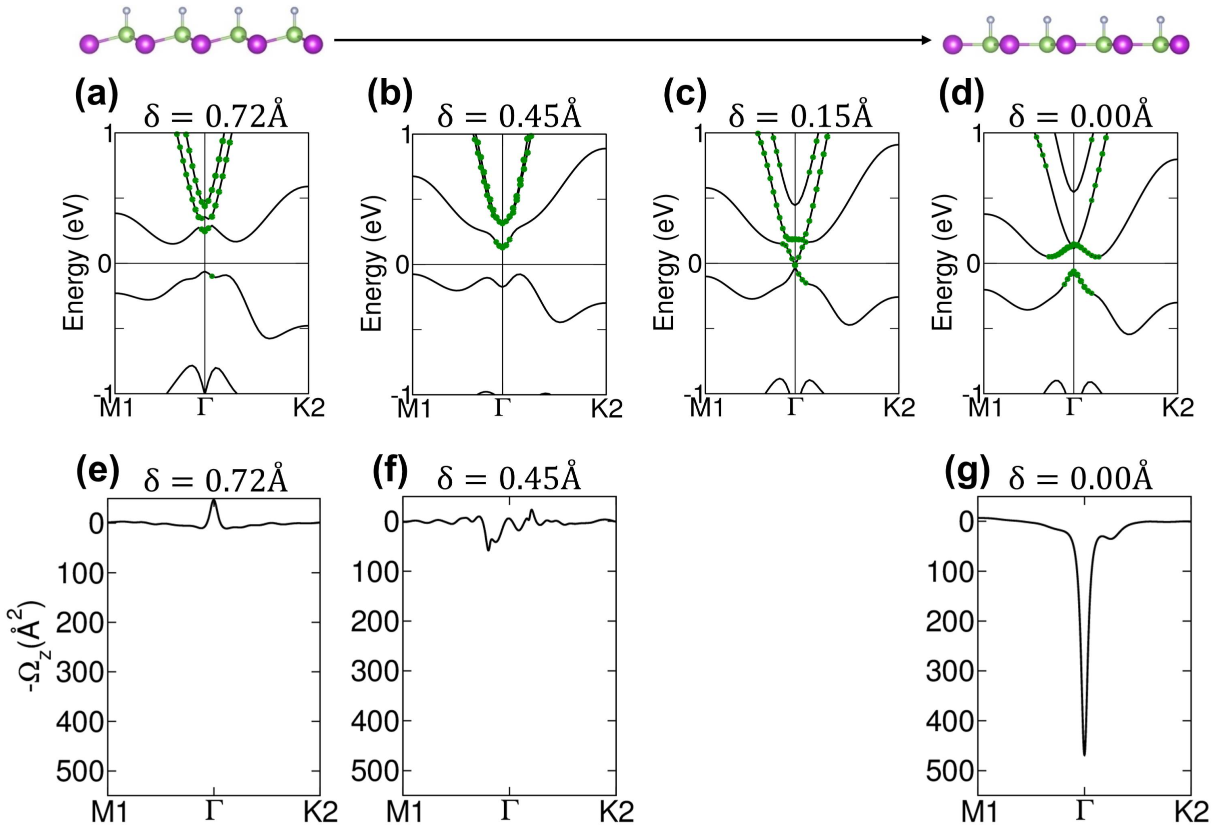

Figure S2. (a)-(d) Band structures for buckled GaBi films with F-adsorbed on Ga at various values of the buckling distance δ, showing the transition to a QAH insulator with a critical point at δ=0.15 Å. These band structures are the same as those shown in Figs. 2(c)-(f) in the main text, and are reproduced here for ease of references. p_x-orbital contribution is shown (solid green circles). (e)-(g) Berry curvatures corresponding to the band structures in panels (a), (b) and (d) are shown in (e), (f), and (g), respectively. Results corresponding to the band structure of panel (c) is not shown as the Berry curvature becomes undefined at the critical point.

III. Topological phase transition as a function of strain

Fig. S3 shows the topological phase transition as strain is varied in a buckled GaBi film with F on Ga. The film is seen to undergo a transition to a QAH insulator at a=5.26 Å,

where the conduction and valence bands are seen to touch each other at Γ.

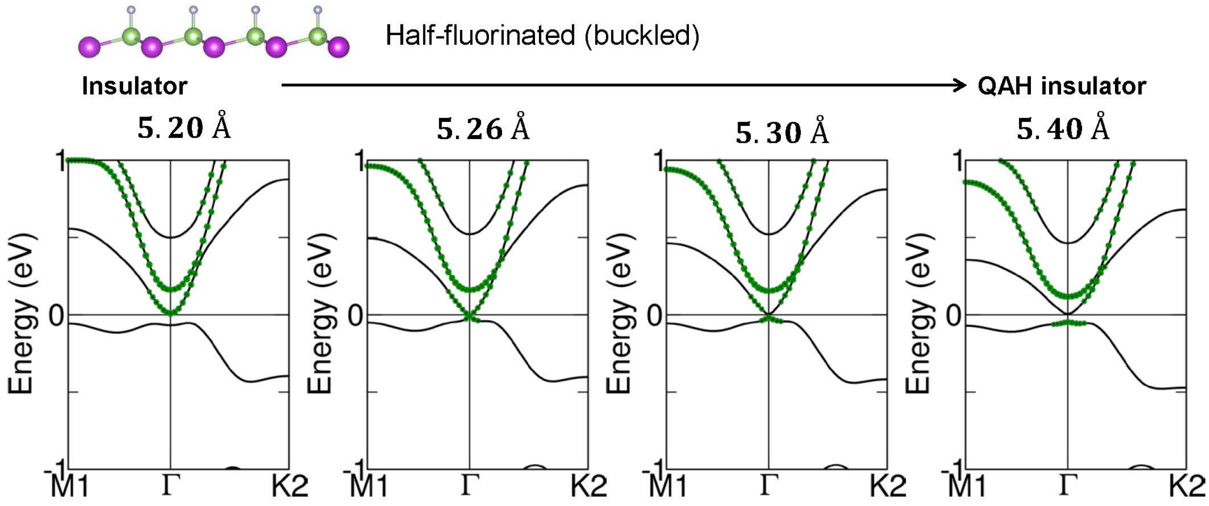

Figure S3. Band structures for a buckled GaBi films with F-adsorbed on Ga at various values of the strain, showing the transition to a QAH insulator with a critical point at a =5.26 Å. p_x-orbital contribution is shown (solid green circles).

IV. Topological phase transition of a planar BiGa film with half-fluorination on Ga

For the planar case, the system transitions from a QAH semi-metal to a QAH insulator at a=4.68Å , see Fig. S4(a). Another phase transition is found as the lattice constant is further increased, with a critical point at 5.325 Å where the band inversion takes place at $K1$, and the film becomes a trivial insulator, see Fig. S4(b).

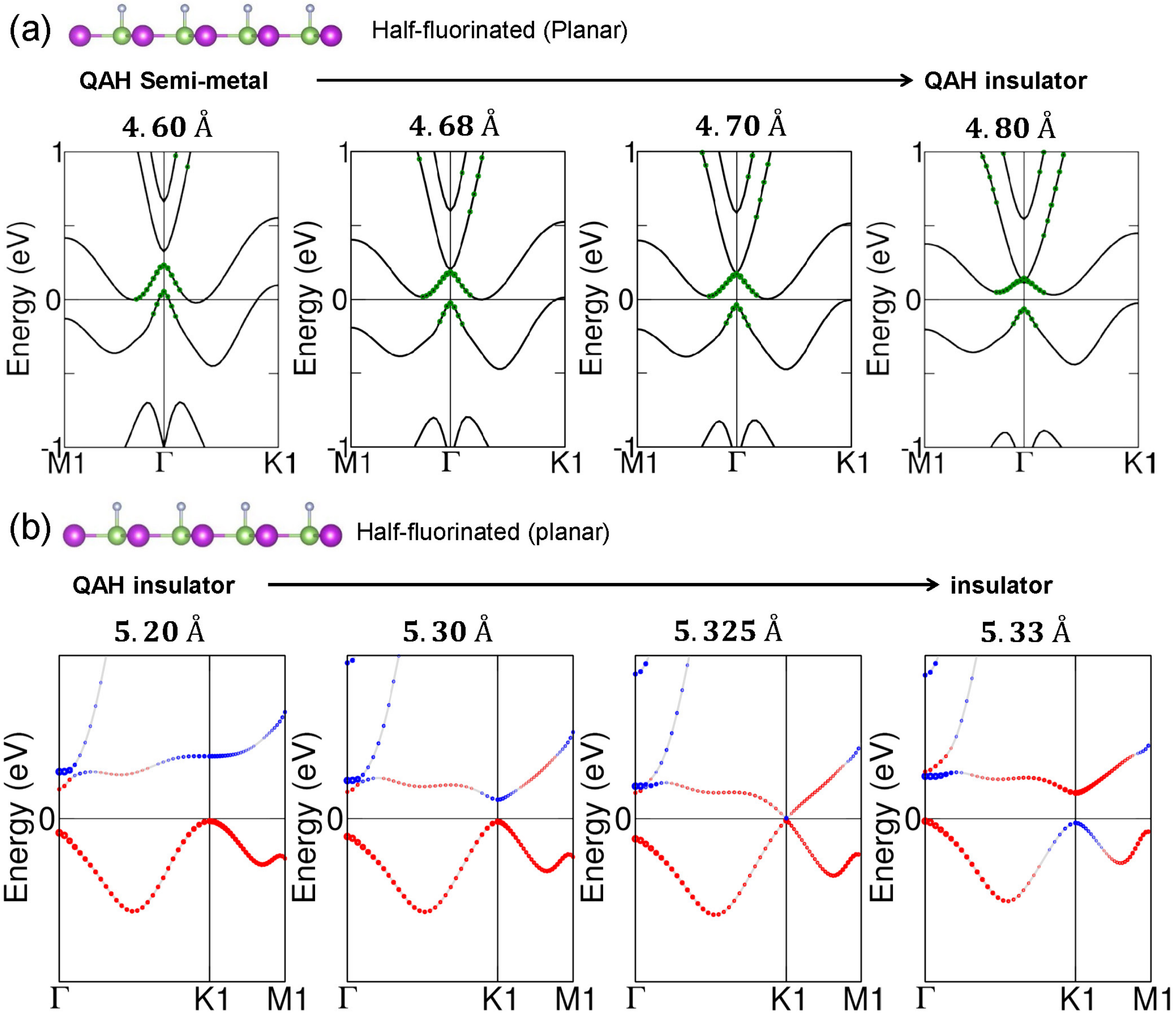

Figure S4. (a) Band structures of planar GaBi films with F-adsorbed on Ga at various values of the lattice constant, showing transition from a QAH semi-metal to a QAH insulator at a=4.68Å. The p_x-orbital contributions are indicated by green circles. (b) Band structures at higher values of the lattice constants showing the transition from a QAH insulator to a trivial insulator. The p_z-orbital contribution is proportional to the size of the circles. The red and blue colors denote different spin polarizations.

V. Further details of band structures of GaBi with F adsorption on Ga

Fig. S5 gives the p_y-orbital contributions for various computed band structures, in addition to the p_x-orbital contributions which were given in Fig. 3 of the main text.

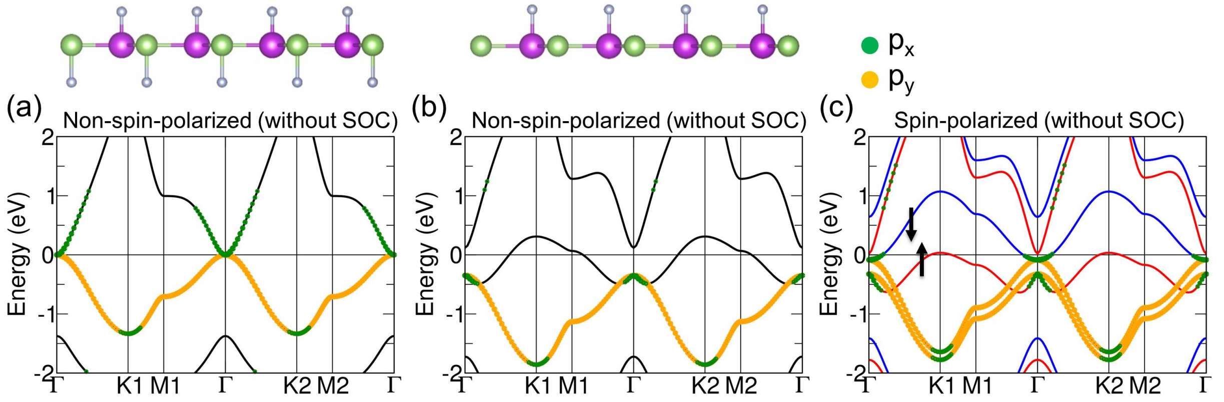

Figure S5. Band structures of GaBi films with F adsorption on Ga: (a) fully-fluorinated, non-spin-polarized film, without SOC; (b) and (c) half-fluorinated film with F on Ga (BiGa-F), without SOC. Non-spin-polarized (b) and spin-polarized (c) band structures. The p_x- and p_y-orbital contributions are labeled by green and orange circles, respectively. In (c) the $+m_z$ and $-m_z$ are, respectively, represented by red and blue lines/circles.

VI. Band structures of other half-halogenated planar BiGa honeycombs

Other halogen group elements (Cl, Br, and I) were also used to create half-halogenations on planar GaBi films. Like the half-hydrogenated and half-fluorinated films, we found that other halogen atoms also prefer to adsorb on Ga rather than Bi atoms. At their equilibrium lattice constants both half-chlorinated and half-brominated films assume a QAH semi-metal phase, whereas the half-iodinated film is trivial. However, the half-iodinated film transitions into a QAH phase under compressive strain. Band structures and the associated equilibrium lattice constants and magnetizations for the various halogenations (F, Cl, Br, and I) are compared in Fig. S6.

	F	Cl	Br	I
Lattice constant of equilibrium state (Å)	$\simeq 4.80$	$\simeq 4.818$	$\simeq 4.824$	$\simeq 4.852$
Magnetization of $\vec{z}$ (μB)	0.76	0.52	0.47	0.26
	QAH insulator	QAH semi-metal	QAH semi-metal	Semi-metal

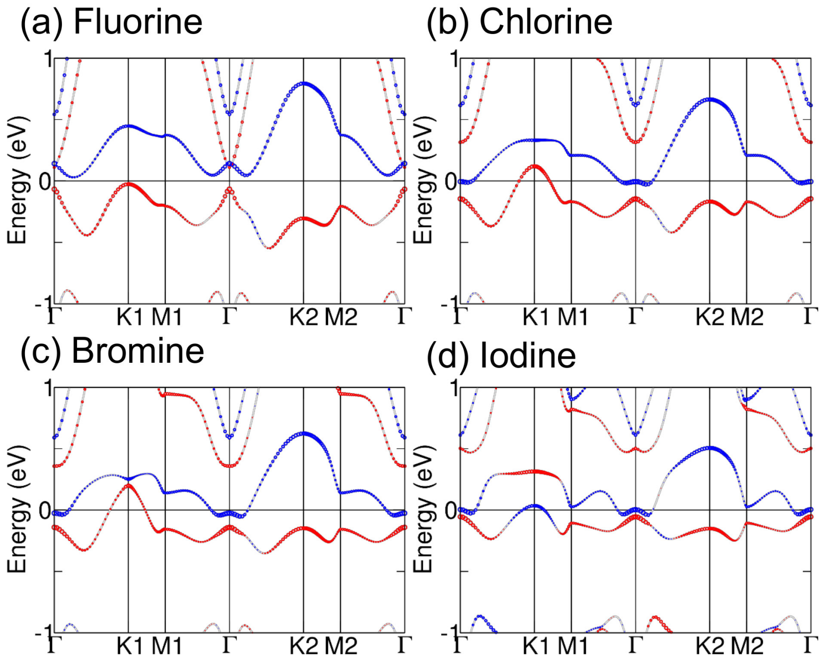

Figure S6. Band structures of planar BiGa honeycombs with halogenation on Ga: (a) half-fluorinated, (b) half-chlorinated, (c) half-brominated, and (d) half-iodinated films at their equilibrium lattice constants, which are given along with the associated magnetic moments per unit cell. All band structures are spin-polarized and include SOC. p_x-orbital contributions are proportional to the size of the circles. Spin-up and spin-down states are shown separately by red and blue lines, respectively.
